# CrEdit: CRISPR mediated multi-loci gene integration in *Saccharomyces cerevisiae*

**DOI:** 10.1186/s12934-015-0288-3

**Published:** 2015-07-07

**Authors:** Carlotta Ronda, Jérôme Maury, Tadas Jakočiu̅nas, Simo Abdessamad Baallal Jacobsen, Susanne Manuela Germann, Scott James Harrison, Irina Borodina, Jay D Keasling, Michael Krogh Jensen, Alex Toftgaard Nielsen

**Affiliations:** The Novo Nordisk Foundation Center for Biosustainability, Technical University of Denmark, Kogle Allé 6, 2970 Hørsholm, Denmark

**Keywords:** Metabolic engineering, CRISPR/Cas9, Genome editing, *Saccharomyces cerevisiae*, Carotenoid production, Genome integrations

## Abstract

**Background:**

One of the bottlenecks in production of biochemicals and pharmaceuticals in *Saccharomyces cerevisiae* is stable and homogeneous expression of pathway genes. Integration of genes into the genome of the production organism is often a preferred option when compared to expression from episomal vectors. Existing approaches for achieving stable simultaneous genome integrations of multiple DNA fragments often result in relatively low integration efficiencies and furthermore rely on the use of selection markers.

**Results:**

Here, we have developed a novel method, CrEdit (CRISPR/Cas9 mediated genome Editing), which utilizes targeted double strand breaks caused by CRISPR/Cas9 to significantly increase the efficiency of homologous integration in order to edit and manipulate genomic DNA. Using CrEdit, the efficiency and locus specificity of targeted genome integrations reach close to 100% for single gene integration using short homology arms down to 60 base pairs both with and without selection. This enables direct and cost efficient inclusion of homology arms in PCR primers. As a proof of concept, a non-native β-carotene pathway was reconstructed in *S. cerevisiae* by simultaneous integration of three pathway genes into individual intergenic genomic sites. Using longer homology arms, we demonstrate highly efficient and locus-specific genome integration even without selection with up to 84% correct clones for simultaneous integration of three gene expression cassettes.

**Conclusions:**

The CrEdit approach enables fast and cost effective genome integration for engineering of *S. cerevisiae*. Since the choice of the targeting sites is flexible, CrEdit is a powerful tool for diverse genome engineering applications.

**Electronic supplementary material:**

The online version of this article (doi:10.1186/s12934-015-0288-3) contains supplementary material, which is available to authorized users.

## Background

The production of bio-based chemicals, fuels, pharmaceuticals and food additives by microbial fermentation is a rapidly growing field. There is an increasing demand for efficient cell factories that enable the production of biofuels and biochemicals from renewable resources at low and competitive cost. The knowledge of genetics, physiology, biochemistry and large-scale fermentation of baker’s yeast *Saccharomyces cerevisiae*, combined with the advent of genome engineering and recombinant DNA technology makes it a preferred host for many industrial bio-based applications, ranging from biofuels and bulk chemicals to nutraceuticals and pharmaceuticals [1–8]. Furthermore, *S. cerevisiae* has the advantage of being easy to manipulate genetically with a range of established cloning and vector systems [6, 9].

Production organisms with multi-enzyme pathways often require precise control of the expression level of the associated genes [2, 5, 10]. Besides regulating promoter strength, the copy number of genes is a critical control point. Both plasmid and genomic integration systems are widely used for heterologous expression of genes in *S. cerevisiae*. Plasmid-based systems typically offer limited control of copy number, and significant segregational instability of plasmids is often observed even during growth in selective medium [10]. It has for example been demonstrated that plasmid-based gene expression is highly heterogeneous, and that both 2µ and CEN/ARS vectors can be difficult to maintain at a stable level within the same cell population [11, 12]. Genomic integration is therefore the preferred alternative to ensure long-term stability and homogeneous expression of genes within a population.

Methods that enable fast, sequential or combinatorial integrations are valuable for metabolic engineering. Several powerful approaches, either plasmid- or PCR-based, have been demonstrated for genome integrations using selection markers. Such methods typically use active recombination systems, such as Cre/LoxP and FLP/FRT, to excise the marker without the need of counter selection [13, 14]. Recently, Jensen et al. developed an efficient set of vectors, the EasyClone vector set, that enables fast and simultaneous multiple integrations of genes into specific “safe sites of insertion” with the possibility of recycling the selective markers [12]. The insertion sites are located between essential elements, which limits the occurrence of chromosomal aberrations due to the lethal effect this would cause [15]. Based on homologous recombination using 500 bp long homology arms, this method results in successful integration into a single site [12]. However, the efficiency of integration decreases when native genes or promoters are present on the fragment to be integrated, or in the case of multiple simultaneous integrations (unpublished results). Jensen et al. reported 44% integration efficiency for simultaneous integration of three heterologous genes at three different loci using selection [12]. Increasing the efficiency of targeted integration without selection is therefore important for accelerating and potentially automating the strain engineering process.

The recent advent of CRISPR/Cas9 for genome engineering has enabled efficient genome editing in different organisms such as bacteria [16], mice [17], plants [18], fruit flies [19], fish [20] and mammalian cells [21–23]. CRISPR/Cas9 has also been applied for targeted single and multiple gene deletions in *S. cerevisiae* by homology-directed repair of double-strand breaks (DSBs) using short oligonucleotides as repair donors, in different strain backgrounds [24–29]. The prevalent DSB repair mechanism in *S. cerevisiae* is native homologous recombination (HR), and the introduction of a DSB has been shown to increase integration of heterologous linear DNA fragments with ends homologous to the DSB site [30, 31]. Harnessing HR for DSB repair, Ryan et al. recently reported the successful integration of a three-part DNA assembly into a single chromosomal locus [26], and Mans et al. performed a complete deletion of the *ACS2* locus in combination with a six-part DNA assembly that resulted in the deletion of the *ACS1* locus [26, 28]. This impressive approach, however, most likely requires additional intrinsic selection pressure, with the simultaneous deletion of these two loci being essential for viability. Furthermore both Horwitz et al. and Jakociunas et al. have recently shown multiplex assembly and integration of multiple parts in three loci, albeit with relatively low efficiencies [27, 29, 32]. Jakociunas et al. have demonstrated the powerful application of the CRISPR/Cas9 system as a tool for metabolic engineering utilizing user-friendly and easy-to-use USER-technology-based gRNA constructs [27]. In order to further expand this existing platform for knock out constructions, we wished to investigate whether CRISPR/Cas9 together with the DNA brick based EasyClone approach could be employed for targeted one-step selection-free integration of multiple genes into the *S. cerevisiae* genome.

Here, we have developed a system, CrEdit (CRISPR/Cas9 mediated genome Editing), which combines the high specificity of CRISPR/Cas9 with the convenient genome engineering tool EasyClone for achieving highly efficient and accurate simultaneous genomic integration of multiple pathway gene expression cassettes in different loci in the genome of *S. cerevisiae*. The gRNA-guided Cas9 endonuclease was used to target gene integration at selected insertion sites, which resulted in up to 100% correct selection-free target integration at the desired locus for the donor DNA. CrEdit also enabled simultaneous and highly efficient integration of three pathway genes involved in the production of β-carotene at three different integration sites located on three different chromosomes.

## Results and discussion

### Construction of the CrEdit system

**Figure 1:**
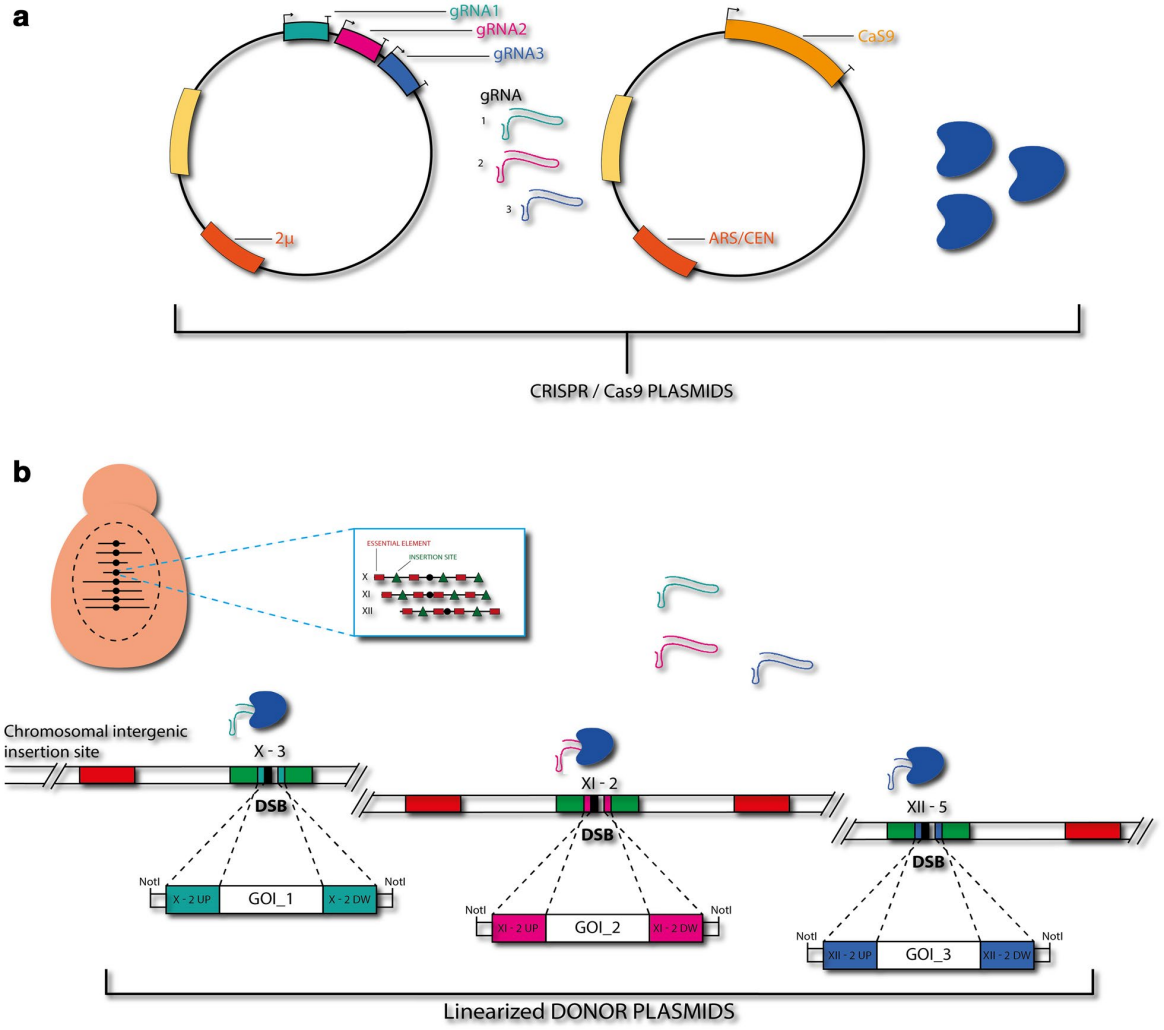
Schematic overview of the CrEdit system. **a** Replicative vectors expressing gRNAs and *cas9*. **b** Targeted DSBs mediated by Cas9 endonuclease activity facilitate the integration of linearized donor plasmids by homologous recombination. Donor plasmids harbor the desired integration sequences flanked by homology arms. Selected intergenic safe harbor sites can be used for simultaneous integration of multiple genomic expression cassettes for pathway engineering.

In order to increase the efficiency of targeted integration into the *S. cerevisiae* genome, we decided to combine the well-characterized genomic integration sites used in the EasyClone system with the RNA-guided endonuclease activity of Cas9. Initially, we tested two different designs for the system. In the first design, Cas9 was expressed from a constitutive promoter, P_*TEF1*_, on an ARS/CEN based vector, while the gRNA that targets Cas9 to the chosen EasyClone integration site was expressed from an episomal 2μ-based vector (Figure 1) [24]. In the second design, Cas9 was under the control of the inducible P_*CUP1*_ promoter and integrated in the genome, and the gRNA supplied on a linearized integrative vector. The first design was chosen for its versatile and recyclable aspects, while the second design was chosen for the possibility of controlling the expression of Cas9 and gRNAs at lower levels. Both types of gRNA carrier plasmids have been designed to enable a fast exchange of the gRNA expression cassettes via USER cloning. Thereby, it is possible to conveniently target a new locus by quick and easy single-step cloning of the gRNA plasmids [27]. Also, the USER-overhang system enables multiplexing of up to five gRNAs on one single plasmid [27].

In this study, we show the use of both genomic and plasmid versions in combination with the donor DNA being provided via EasyClone integration plasmids. The donor DNA can contain up to two promoter-gene-terminator sequences, a selection marker flanked with loxP sites, and homology arms for homologous recombination at the defined insertion sites of the EasyClone system [12]. Importantly, for targeting integration site X-3, the sequence of the donor integration plasmid was modified by eliminating the PAM site (protospacer-adjacent motif, i.e. three nucleotides necessary for Cas9 recognition), since the PAM is located on a donor homology arm. This design prevents Cas9 from cutting the target sequence once successful integration has occurred. In the other sites used, the PAM site is located within a section of the genome that is deleted by the successful integration event of the two interspaced homology arms. Since the PAM sequence is removed in case of completed integration, this might have an additional positive effect on obtaining correct transformants, since Cas9 keeps cutting in cells where integration was not successful. Thereby the DSB fails to be repaired, which is lethal for the cells [24].

### Targeted single genomic integration of *tHMG1*

**Figure 2:**
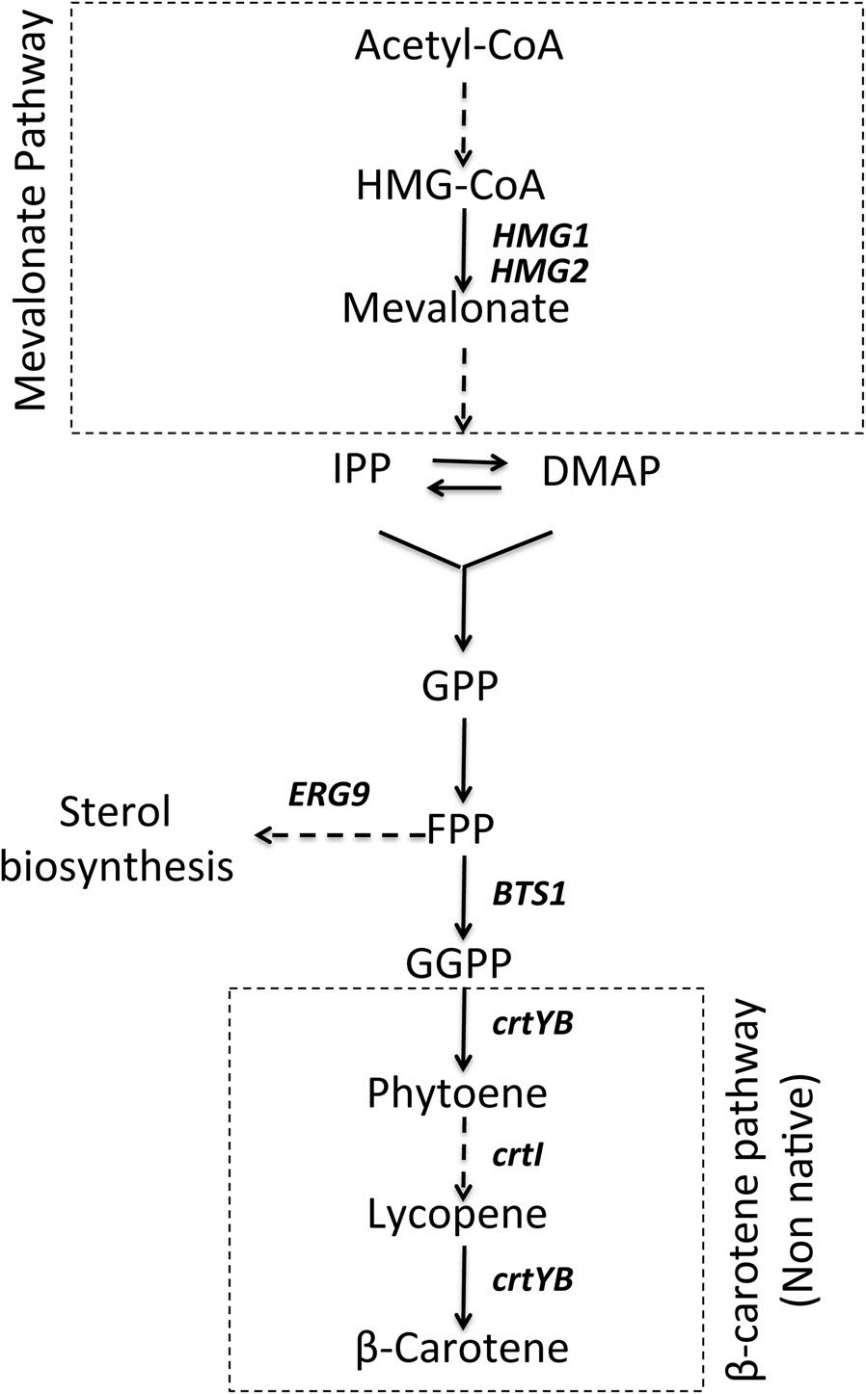
Overview of the biosynthetic pathway for β-carotene production. The carotenoid biosynthetic pathway can be reconstructed in *S. cerevisiae* by overexpression of the native GGPP synthase encoded by *BTS1*, and co-overexpression of the non-native bifunctional phytoene synthase/lycopene cyclase encoded by *crtYB*, and phytoene desaturase encoded by *crtI* of *X. dendrorhous*. *HMG1* encodes the major HMG-CoA reductase activity in *S. cerevisiae*. *ERG9* encodes a farnesyl-diphosphate farnesyl transferase (squalene synthase) that acts in the sterol biosynthesis pathway. *IPP* isopentenyl diphosphate, *DMAP* dimethylallyl diphosphate, *GPP* geranyl diphosphate, *FPP* farnesyl diphosphate, *GGPP* geranylgeranyl diphosphate.

As a proof of concept for the applicability of CrEdit for metabolic engineering, we used the well-established carotenoid biosynthetic pathway as a model. Carotenoids are part of the diverse group of natural compounds called isoprenoids, and are synthesized from precursors derived from the native mevalonic acid (MVA) pathway (Figure 2). The *tHMG1* gene encodes a truncated HMG-CoA reductase, which has been shown to increase carbon flux through the pathway, leading to increased isoprenoid and carotenoid production [33, 34]. Therefore, we initially focused on introducing one copy of the *tHMG1* overexpression cassette into the *S. cerevisiae* genome.

**Figure 3:**
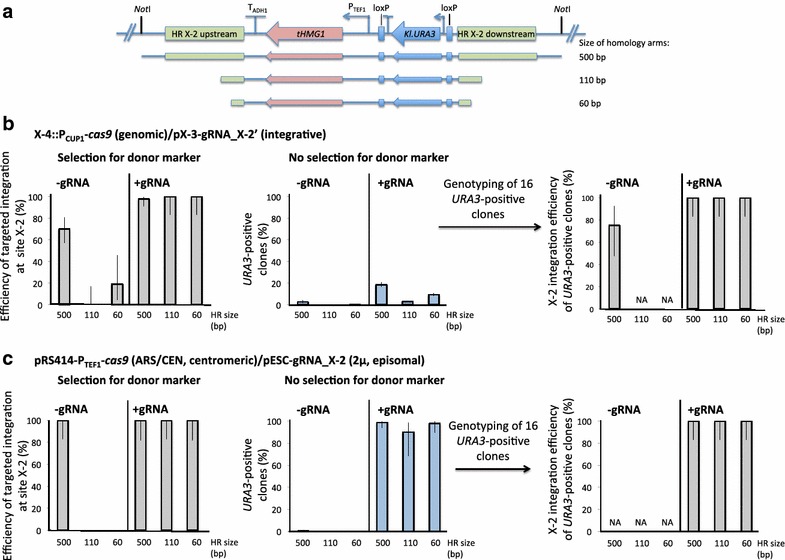
Integration efficiency of *tHMG1* at locus X-2 using different lengths of homology arms. **a** Overview of the donor DNA fragment bearing *tHMG1* with differently sized homology arms. **b** Integration efficiency of the CrEdit system with genomic inducible Cas9 and integrative gRNA. *S. cerevisiae* strain ST1011 harboring P_*CUP1*_-*cas9* was induced with Cu^2+^ 2 h prior to transformation start, and then co-transformed with (*left*, –gRNA) linearized empty vector pCfB257 and linearized donor DNA encoding *tHMG1* (for details of donor DNA see Additional file 1), or (*right*, +gRNA) the linearized integrative gRNA vector pCfB2831 targeting X-2 and linearized donor DNA encoding *tHMG1.* *Left panel* Efficiency of targeted integration at site X-2 when selecting for donor DNA after transformation. *Middle panel* Efficiency of marker gene integration when not selecting for donor DNA after transformation. *Right panel* Frequency of correct integration at site X-2 determined by genotyping of URA^+^ colonies. **c** Integration efficiency of the CrEdit system with plasmid-based Cas9 and gRNA. *S. cerevisiae* strain TC-3 harboring P_*TEF1*_-*cas9* on the centromeric plasmid pCfB1767 was co-transformed with (*left*, −gRNA) empty vector pCfB2999 and linearized donor DNA encoding *tHMG1*, or (*right*, +gRNA) the episomal gRNA vector pCfB3020 targeting X-2 and linearized donor DNA encoding *tHMG1.* *Left panel* Efficiency of targeted integration at site X-2 when selecting for donor DNA after transformation. *Middle panel* Efficiency of marker gene integration when not selecting for donor DNA after transformation. *Right panel* Frequency of correct integration at site X-2 determined by genotyping of URA^+^ colonies. Only +gRNA colonies were analyzed since no *URA* ^+^ clones were obtained in the absence of gRNA. The experiment was repeated twice and error bars represent 95% confidence intervals. *NA* not analyzed.

In order to test the efficiency of the two different CrEdit designs, we decided to test single integration of donor DNA with differently sized homology arms. As donor we used an EasyClone integrative plasmid containing *tHMG1* with homology arms specific for intergenic site X-2 (Figure 3a) [15]. The integration efficiencies of all experiments are shown in Additional file 1: Table S1. We first tested the integration efficiency of using integrative gRNA in combination with a *S. cerevisiae* strain harboring genomic *Cas9* under the control of the P_*CUP1*_ promoter. Cas9 expression was induced by addition of Cu^2+^ 2 h before transformation. We then co-transformed this Cas9-expressing strain with the specific donor DNA carrying *tHMG1* with homology arms of 500, 110 or 60 bp length for site X-2, and the integrative gRNA targeting site X-2. An empty vector backbone without gRNA was used as a control. The resulting transformants were plated onto medium selecting for Cas9, the gRNA and the donor selection marker. We then analyzed the genotype of at least 16 colonies per condition to check for correct insertion at site X-2. When relying solely on intrinsic homologous recombination, the measured efficiency of correct integration at site X-2 was 70% with homology arms of approximately 500 bp (Figure 3b, left panel, −gRNA). As expected, the efficiency of correct integration was found to decrease significantly when using shorter arms with lengths of either 110 or 60 bp (Figure 3b, left panel, −gRNA). However, when the gRNA targeting X-2 was expressed, close to 100% successful integration was obtained at site X-2, regardless of the length of the homology arms (Figure 3b, left panel, +gRNA). Interestingly, when using the plasmid-based gRNA/Cas9 system and in the absence of gRNA, 100% correct integrants could only be obtained using 500 bp homology arms. Furthermore, and only in that condition, a low number of transformants was obtained on plates, which points towards a negative effect of *cas9* expression on cells when expressed from the constitutive strong *TEF1* promoter and in the absence of gRNA. Ryan et al. reported a decreased fitness of yeast strains expressing *cas9* from the strong *TDH3* promoter [26], while Mans et al. reported that the constitutive expression of *cas9* from the genome and the *TEF1* promoter does not affect the maximal specific growth rate on glucose based synthetic media [28]. In light of these results, a more detailed study of the impact of *cas9* expression levels on yeast cell physiology and especially the HR machinery is of interest. Still, 100% correct integrants were obtained in the presence of gRNA for all sizes of homology arms (Figure 3c, left panel), suggesting that the plasmid-based gRNA/Cas9 system also is very efficient.

In conclusion, we show that the DSB created by the guide RNA-targeted Cas9 endonuclease is instrumental for correct integration at a significantly higher efficiency than what can be achieved solely by endogenous homologous recombination. The lower efficiency observed in absence of CRISPR/Cas9 is possibly due to the fact that native genes tend to recombine at the native locus due to the large homology region. Also, expression cassettes might integrate elsewhere in the genome possibly via break-induced replication (BIR), thus creating strains where it becomes difficult to localize the gene of interest. The targeted DSB created by Cas9 likely boosts HR at the desired integration site.

### Targeted genomic integration without selective pressure

Because of the high efficiency observed for integration of *tHMG1*, we investigated if integration of this gene expression cassette could be performed even without applying selection pressure for the donor DNA marker *Kl.URA3*. We repeated the integration experiment described above, however this time plating the transformants on medium only selecting for gRNA and Cas9. When the plasmid-based gRNA/Cas9 CrEdit system was used, 99, 90, and 98% efficiency of integration of the marker gene was observed for 500, 110 and 60 bp homology arms, respectively (Figure 3c, middle panel, +gRNA). The PCR analysis at locus X-2 for the resulting *Kl.URA3*-positive clones showed 100% correct integration into site X-2 for all tested sizes of homology arms (Figure 3c, right panel, +gRNA). However, when using the genomic CrEdit system with induced P_*CUP1*_-*cas9*, only 19, 3 and 9% integration efficiency were achieved for 500, 110 and 60 bp homology arms, respectively (Figure 3b, middle panel, +gRNA). Despite the lower integration efficiency, PCR analysis of the resulting *Kl.URA3*-positive clones showed 100% correct integration into site X-2 for all tested sizes of homology arms (Figure 3b, right panel, +gRNA). When the empty vector (−gRNA) was included in the transformation, the efficiency of marker integration was close to zero in all cases, independent on the length of the homology arms (Figure 3b, c, middle panels, −gRNA). In the case of genomic *cas9* and long 500 bp homology arms, the genotyping of 16 *Kl.URA3*-positive clones showed approximately 75% correct integration at site X-2 (Figure 3b, right panel, −gRNA). Differences in promoters between the systems, and the time-limited induction of *cas9* by the *CUP1* promoter in our experimental set-up (2 h prior to transformation) may lead to lower levels of Cas9 at transformation start compared to the plasmid-based system where *cas9* is under the control of the constitutive *TEF1* promoter on a centromeric plasmid. In conclusion, the highest efficiency of both selection- and non-selection based genomic integration was achieved when both gRNA and *cas9* were expressed from plasmids, and we therefore chose this to be the final configuration of the CrEdit system (Figure 1).

### Targeted simultaneous multi-loci integration of three carotenogenic pathway genes

In order to speed up the strain construction process, it is often desirable to simultaneously insert multiple genes into the genome. After having achieved highly efficient insertion of *tHMG1* into intergenic site X-2 using the CrEdit method, we tested simultaneous integration of multiple genes into the genome of *S. cerevisiae*. As a proof of concept, we attempted to introduce the non-native production of carotenoids in *S. cerevisiae* via expression of the two heterologous genes *crtYB* and *crtI* of *X*. *dendrorhous* combined with overexpression of *S. cerevisiae* geranylgeranyl diphosphate (GGPP) synthase encoded by *BTS1* [35]. The gene *crtYB* encodes a bi-functional enzyme with phytoene synthase and lycopene cyclase activity, while *crtI* encodes a phytoene desaturase [36].

**Figure 4:**
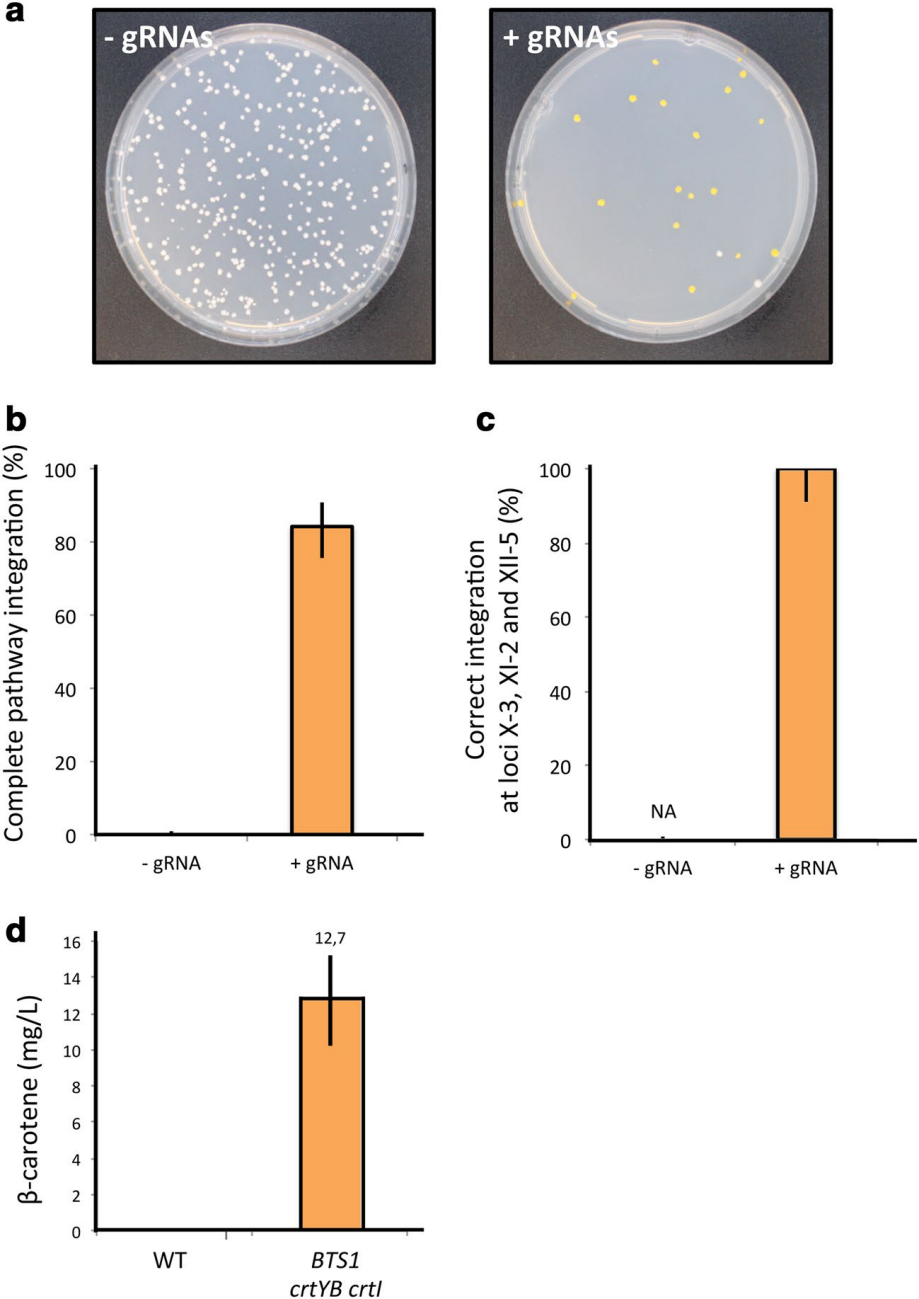
Multiple simultaneous integration of three β-carotene pathway genes. **a** Picture of colonies after simultaneous integration of *BTS1*, *crtYB* and *crtI* on plates without selection. *S. cerevisiae* (TC-3) harboring a centromeric plasmid constitutively expressing *cas9* was co-transformed with: *left* empty vector control and linearized donor DNAs encoding *BTS1*, *crtYB* and *crtI*. *Right* gRNA vector expressing three gRNAs targeting intergenic loci XII-5, XI-2 and X-3, and linearized donor DNAs encoding *BTS1*, *crtYB* and *crtI.* Colonies with successful pathway integration accumulate β-carotene, resulting in an orange pigmentation. **b** Percentage of complete pathway integration with and without the expression of targeting gRNAs. **c** Frequency of correct simultaneous integration of the three genes *BTS1*, *crtYB*, and *crtI* at the specific intergenic loci XII-5, XI-2 and X-3, respectively, determined by genotyping. **d** HPLC analysis of β-carotene production of three independent orange colonies and a non-producing strain as control (CEN.PK113-7D). The experiment was repeated twice and *error bars* in all *panels* represent 95% confidence intervals.

Using the plasmid-based CrEdit system, cells expressing Cas9 were simultaneously transformed with three different large EasyClone donor DNAs for integration of P_*TDH3*_-*crtI* (6.6 kb), P_*TEF1*_-*crtYB* (5.8 kb), and P_*PGK1*_-*BTS1* (5.1 kb) into three intergenic sites X-3, XI-2, and XII-5 situated on different chromosomes, using 500 bp homology arms. The cells were co-transformed with one episomal vector expressing the three gRNAs targeting these three sites, or with the empty vector for the −gRNA control. Transformants were plated on media selecting only for Cas9 and gRNA expressing plasmids. We observed that 84% of the derived colonies presented orange pigment formation when the gRNAs were present, indicating complete β-carotene pathway integration. In contrast, we only observed white colonies when the gRNAs were absent, indicating that no correct triple integration had been achieved (Figure 4a). All colonies were then replicated on single drop-out plates in order to screen for the integration of the three independent selection marker genes. As expected, all orange colonies were positive for all the three marker genes (Figure 4b, left panel). We subsequently tested the genotype of 32 orange colonies at the three expected integration sites, and observed 100% correct integration, thereby confirming complete pathway assembly (Figure 4b, right panel). In addition, we measured β-carotene levels by HPLC in three confirmed clones, and demonstrated that 12.7 ± 2.5 mg L^−1^ β-carotene was produced (Figure 4c). This proves the ability of the CrEdit system to simultaneously integrate three large DNA fragments with surprisingly high efficiency (84%) at the correct loci even without selection pressure. As for comparison, simultaneous integration of three genes has previously been demonstrated with 44% efficiency when relying on native HR alone and when applying selective pressure [12]. It was furthermore attempted to repeat the multi-loci pathway integration using short homology arms (60 bp) to investigate if we could simply use PCR products directly as donors for the multiplex integration. However, no viable colonies grew on the plates even after 1 week of incubation (data not shown), indicating that longer homology arms are beneficial for multiplex genome integrations. We assume this may due to the fact that multi-loci pathway integration is quite demanding with regards to coordinated repair activity, and long homology arms are easier to utilize for the native yeast HR machinery, thereby enabling correct simultaneous integration at multiple loci.

The results obtained for simultaneous integration of three genes (*BTS1*, *crtYB* and *crtI*) show the ability of the CrEdit system to insert very large fragments (up to 17.5 kb in this study) without the need for a selection marker, which is very attractive for industrial metabolic engineering applications. Industrial strains are often prototrophic, and/or diploid or even polyploid, thus making the use of auxotrophic markers challenging. Furthermore, even for haploid auxotrophic strains, the limited number of available selection markers typically necessitates recycling of the markers. Several systems can be used for looping out genetic elements, including the Cre-LoxP and FRT/FLP systems [12–14]. Such methods are not only time consuming but can also leave scars, which can cause genome instability and rearrangements in recombinant strains [37]. Importantly, CrEdit enables selection-free and scarless integration of desired DNA sequences, thereby limiting the risk for strain instability while significantly speeding up strain engineering. Moreover, CrEdit is a versatile genome engineering tool, since the design of novel gRNAs for alternative integration sites can be easily achieved using for example the recently developed in silico gRNA selection tool, CRISPy, which minimizes the potential risk of off-target effects of Cas9 activity [23, 27].

Recently, high efficiencies using CRISPR/Cas9 system for genome integration have been reported, but most systems either still rely on selective pressure or, if selection was not applied, only short DNA sequences were inserted [25, 26]. In the recent work by Horwitz et al., an 11 gene pathway was integrated via 6-part integration, however only very low efficiency was observed [32]. Stovicek et al. also demonstrated successful multi-part assembly at three different loci, yet with relatively low efficiencies [29]. In contrast, CrEdit is a versatile system for achieving high efficiency of single and multiple simultaneous integrations without the need for selection (when long homology arms are used). The CrEdit system was designed in a way that the PAM sequence is eliminated upon successful integration. The continued cutting of the wild-type DNA is thereby possibly contributing to the very high efficiency of integration [24].

### Further engineering of carotenoid production

A significant amount of work is available on engineering organisms for production of carotenoids [38–41], and in recent years a biosustainable and economically attractive production of β-carotene has been achieved [34, 42]. In an attempt to further boost β-carotene production, we integrated the β-carotene pathway in *S. cerevisiae* strains bearing genetic modifications previously reported as being beneficial for the flux to the mevalonate pathway. We therefore performed the multi-loci integration experiment in a CEN.PK strain carrying a down-regulated version of the squalene synthase *ERG9* gene (*erg9*::Δ-220–176). In this strain, a deletion of an upstream section of the promoter causes lower *ERG9* transcript and protein levels, thereby reducing the flux towards the competing endogenous sterol biosynthetic pathway [27, 43]. We also transformed a CEN.PK strain that carried both the *erg9*::Δ-220–176 modification and an overexpression of *tHMG1*. Orange colonies producing carotenoids were obtained with high efficiency in both genetic backgrounds (Additional file 1: Figure S1). It was also observed that these latter strains were clearly less orange compared to the unmodified CEN.PK strain only expressing the β-carotene pathway (compare Figure 4 and Additional file 1: Figure S1). β-carotene concentrations were measured and it was shown that the additional genetic modifications did not lead to an increase in the β-carotene levels. A significant decrease in β-carotene concentration was even observed for the strain bearing both *erg9*::Δ-220–176 and the overexpression of *tHMG1* (Additional file 1: Figure S1). Indeed, Verwaal et al. have shown that the desaturation of phytoene, catalyzed by CrtI, is a rate-limiting step in carotenoid production, and that an increase of the total carotenoid accumulation is largely caused by a significant increase of this precursor [35]. As phytoene is color-less, it is expected that its accumulation in the strains improved for precursor availability results in the less intense coloration of the yeast colonies. In order to avoid this precursor accumulation, it may be possible to further boost the expression of *crtI* by integrating this pathway gene in more copies [35].

## Conclusion

In summary, we were able to demonstrate the ability of the CrEdit system to simultaneously integrate up to three large DNA fragments with high efficiency even without selective pressure into different genetic backgrounds, supporting the strength and robustness of the method.

CrEdit combines the stability and versatility of the EasyClone vector system with the precision and efficiency of CRISPR/Cas9, thereby significantly increasing the efficiency of genome integrations in *S. cerevisiae*. We demonstrate how this system can be used for simultaneous integration of multiple genes with high efficiency, even without selection for donor DNA. CrEdit is also very efficient in integrating large fragments at single loci using short homology arms of 60 bp that can be included in PCR primers. This facilitates quick and easy exchange from one integration site to another. A further advantage of the primer-based preparation of donor DNA is that the PAM recognition site can easily be removed from the short homology arms. Provided that a suitable PAM sequence is present at the genomic site of interest, the system can easily be developed for other genome engineering applications, such as combining integrations with gene deletions, defined site-specific mutagenesis, gene replacements, promoter exchange, protein domain swapping, in a scarless and selection-free manner. We therefore believe that CrEdit will be a valuable genome engineering tool to facilitate fast and cost-effective production strain engineering.

## Methods

### Strains and media

*Saccharomyces cerevisiae* CEN.PK strains were obtained from Peter Kötter (Johann Wolfgang Goethe-University Frankfurt, Germany). All yeast strains used in this study were derivatives of CEN.PK (Additional file 1: Table S2). All standard cloning was carried out using *E. coli* strain DH5alpha. Media and standard genetic techniques used for manipulating yeast strains were performed as previously described [44]. Synthetic complete medium as well as drop-out media and agar plates were prepared using premixed drop-out powders (Sigma-Aldrich). All chemicals were obtained from Sigma-Aldrich. *Escherichia coli* transformants were grown in standard Luria–Bertani (LB) medium containing 100 µg mL^−1^ ampicillin.

### Construction of plasmids for single targeted integration

All plasmids are described in Additional file 1: Table S3, and all gRNA sequences are listed in the Additional file 1 as well. Construction of expression plasmids used as donor DNA for integration is explained in detail in the Additional file 1. For design of all gRNA target sequences, the overall design was based on DiCarlo et al. [24] (Additional file 1: Table S4), and for designing the target sequence the program CRISPy was used [23, 27]. gRNA plasmid pCfB2831 used for integrating the gRNA X-2′ (targeting site X-2) into chromosomal site X-3 [15] was constructed by amplifying a gRNA expression cassette (ordered from Integrated DNA Technologies as gBlock), gRNA_X-2′ (Additional file 1: Table S5), with primers PR-10735/PR-10736 (Additional file 1: Table S6), and subsequent USER cloning into *Asi*SI/*Nb.Bsm*I-digested pCfB257 according to Jensen et al. [12]. To construct the episomal gRNA plasmid pTAJAK-76 (targeting site X-2), a backbone-cloning vector was created for USER cloning of the gRNA expression cassettes by amplifying and re-ligating pESC-LEU with TJOS-97F and TJOS-97R. Secondly, the resulting vector was amplified using the primers TJOS-108 and TJOS-102R in order to remove the *KlLEU2* marker. The NatMXsyn marker was then amplified from pCfB2180 (GeneArt) with the primers TJOS-106F and TJOS-106R, and USER-cloned into the vector lacking the *KlLEU2* marker, resulting in plasmid pTAJAK-71. Finally, to target site X-2 [15] with Cas9, a gRNA expression cassette was ordered from Integrated DNA Technologies as gBlock, gRNA_X-2 (Additional file 1: Table S5), and amplified with following primers: TJOS-62, TJOS-65. Amplified gRNA was USER cloned into pTAJAK-71, which was previously digested with *Asi*SI/*Nb.Bsm*I, resulting in the plasmid pTAJAK-76.

### Construction of plasmids carrying multiple gRNAs

First, a backbone-cloning vector was created for USER cloning of the gRNA expression cassettes by amplifying and re-ligating pESC-LEU with TJOS-97F and TJOS-97R. Secondly, the resulting vector was amplified using the primers TJOS-108 and TJOS-102R in order to remove the *KlLEU2* marker. The KanMXsyn marker was then amplified from pCfB2179 (GeneArt) with the primers TJOS-106F and TJOS-106R, and cloned into the vector lacking the *KlLEU2* marker, resulting in plasmid pTAJAK-72. Finally, to target the sites X-3, XI-2 and XII-5 [15] with Cas9, gRNA expression cassettes [24] were ordered from Integrated DNA Technologies as gBlocks (gRNA sequences are listed in Additional file 1: Table S4) and amplified with following primers: TJOS-62/TJOS-66 (gRNA_X-3); TJOS-63/TJOS-67 (gRNA_XI-2) and TJOS-64/TJOS-65 (gRNA_XII-5). Amplified gRNAs were USER cloned into pTAJAK-72, which was previously digested with *Asi*SI/*Nb.Bsm*I, resulting in the plasmid pTAJAK-92 according to Ref. [27].

### Transformation protocol for single integration

Plasmids were transformed into *S. cerevisiae* cells using the lithium acetate transformation protocol [45]. Initially, P_*CUP1*_-*cas9* was integrated into EasyClone site X-4 by transforming CEN.PK102-5B with the integrative vector pCFB1129 resulting in strain ST1011 (Additional file 1: Table S3). When transforming strain ST1011, Cas9 transcription was induced by adding 200 µM CuSO_4_ 2 h before harvesting the cells for transformation. Prior to transformation, donor DNA was prepared as follows. For 500 bp homology arms, the integrative vector pCfB772 was digested by *Not*I and column-purified (Nucelospin Gel and PCR cleanup kit, Macherey Nagel). For shorter homology arms, pCfB772 was amplified by PCR using primer sets PR-9706/PR-9707 (110 bp) or PR-9704/PR-9705 (60 bp), *Dpn*I-treated and resolved on 1% agarose gel containing SYBR^®^-SAFE (Invitrogen) and purified using NucleoSpin^®^ Gel and PCR Clean-up kit (Macherey Nagel). For single integration, 1 µg donor DNA and 1 µg *Not*I-linearized integrative gRNA plasmid or 500 ng undigested episomal gRNA plasmid was co-transformed into competent yeast cells. Cells were plated on media that selected for the presence of the gRNA (*KlLEU2*) and Cas9 (*SpHIS5*), and optionally donor marker (*KlURA3*) where stated. When colonies appeared, the transformation plates were replicated on selective plates (SC-LEU, SC-URA, SC-HIS) to screen for colonies with integrated selection markers. Correct integration at the specific genomic locus was verified by colony PCR with following primers: PR-2221/PR-901 (X-2: P_*TEF1*_-*tHMG1*).

### Transformation protocol for multiple integration of carotenoid pathway

To simultaneously integrate three genes required for carotene production, 3 μg of each carrier plasmid (pTAJAK-94, pTAJAK-95, pTAJAK-12) were linearized by *NotI* digestion. *S. cerevisiae* strain TC-3 [27] was co-transformed with these linearized donor plasmids plus 1 μg of triple gRNA plasmid pTAJAK-92. Cells were plated on media that selected for the presence of the gRNA (kanMX) and Cas9 (*TRP1*) plasmids. When colonies appeared, the transformation plates were replicated on selective plates (SC-LEU, SC-URA, SC-HIS) to screen for colonies with integrated selection markers. To screen for correct integrations to the expected loci of carotene genes, colony PCR was performed with following primers: PR-2221/PR-903 (X-3: P_TDH3_-*crtI*); PR-2221/PR-909 (XI-2: P_*TEF1*_-*crtYB*); PR-2221/PR-899 (XII-5: P_PGK1_-*BTS1*). The experiment was carried out in triplicate, and statistical analysis (one-tailed Student’s *t* test) was performed on the complete data set. Multiple integration of carotenoid pathway was further performed in strains TC-23 and ST3450, according to the protocol mentioned above. Strain TC-23 harbors a *erg9*::Δ-220–176 genetic modification [30]. Strain ST3450 was obtained by transforming *S. cerevisiae* strain TC-23 with a *Not*I linearized pCfB2996 and transformants were selected on medium containing nourseothricin. Strain ST3450 therefore harbors *erg9*::Δ-220–176 and a copy of P_*TEF1*_-t*HMG1* integrated at chromosome locus X-2.

### β-Carotene quantification

Three independent orange colonies from *S. cerevisiae* TC-3 containing the three expression cassettes for *BTS1*, *crtYB and crtI* were used to inoculate test tubes containing 4 mL of drop out medium per well. As a reference, a colony of *S. cerevisiae* CEN. PK 113-7D was inoculated in the same conditions, and all cells were cultivated at 30°C with 300 r.p.m shaking. After approximately 48 h of cultivation, 3.5 mL of cultivation broth was centrifuged for 5 min at 4,000 rpm. Then supernatants were discarded and cell pellets resuspended in 0.2 mL of milliQ water. Cell suspensions were transferred to screw-cap tubes, suitable for subsequent cell breakage in a Precellys homogenizer. Glass beads and 1 mL of hexane were added to the cell suspension and cells were mechanically lysed for four cycles, each of 20 s at 6,500 rpm. Tubes were placed on ice for 1 min in between each lysis cycle. Subsequently, tubes were centrifuged for 5 min at 10,000 rpm to separate cell debris, aqueous and solvent fractions. The hexane fraction was collected in glass vials. Hexane was then evaporated in a rotary evaporator, under vacuum, and dry extracts were re-dissolved in 1 mL of ethanol 99%. Extracts were then analysed by LC–MS. LC–MS data was collected on Orbitrap Fusion equipped with a Dionex brand Ultimate 3000 UHPLC pumping system (ThermoFisher Scientific, Waltham, MA, USA). Samples were held in the autosampler at a temperature of 10.0°C during the analysis. 2 μL injections of the sample were made onto a Supelco Discovery HS F5-3 HPLC column, with a 3 μm particle size, 2.1 mm i.d. and 150 mm long. The column was held at a temperature of 30.0°C. The solvent system used was Solvent A “Water with 0.1% formic acid” and Solvent B “Acetonitrile with 0.1% formic acid”. The flow rate was 1.000 mL min^−1^ with an initial solvent composition of %A = 75, %B = 25.0 held until 3.0 min, the solvent composition was then changed following a linear gradient until it reached %A = 0.0 and %B = 100.0 at 15.0 min. This was continued until 20 min, when the solvent was returned to the initial conditions and the column was re-equilibrated until 25 min. The column eluent flowed directly into the Heated ESI probe of the MS which was held at 325°C and a voltage of 3,500 V. Profile data was collected in positive ion mode with resolution setting of 30K and scan range (m/z) = 50–600. The other MS settings were as follows, sheath gas flow rate of 60 units, Aux gas flow rate of 20 units, sweep gas flow rate of 5 units, ion transfer tube temp was 380°C, maximum injection time of 100 ms, S-lens RF level = 60 V, using 1 Microscans and AGC target = 200,000 counts.

## Additional file

Additional file 1:
**Figure S1**. Showing efficiency of single step integration of the beta-carotenoid pathway in different strain backgrounds. Additionally, the file includes supplementary methods. **Table S1**. Efficiency of targeted integration using CrEdit. **Table S2**. List of strains used. **Table S3**. List of plasmids used. **Table S4**. gRNA sequences. **Table S5**. DNA and BioBricks and gBlocks. **Table S6**. Primers used in this study.

## Abbreviations

CRISPR: clustered regularly interspaced short palindromic repeats
PAM: protospacer adjacent motif
FRT: flippase recognition target
FLP: flippase
DSB: double strand break
HR: homologous recombination
gRNA: guide RNA
Kl.URA3: *Kluyveromyces lactis**URA3* gene
Cre: cyclization recombinase
