# Supplementary material for: CrEdit: CRISPR mediated multi-loci gene integration in Saccharomyces cerevisiae
Source: Microb Cell Fact. 2015 Jul 7;14:97. doi: 10.1186/s12934-015-0288-3 (PMC4492099; doi:10.1186/s12934-015-0288-3)
Supplement: Additional file 1: — Figure S1. Showing efficiency of single step integration of the beta-carotenoid pathway in different strain backgrounds. Additionally, the file includes supplementary methods. Table S1. Efficiency of targeted integration using CrEdit. Table S2. List of strains used. Table S3. List of plasmids used. Table S4. gRNA sequences. Table S5. DNA and BioBricks and gBlocks. Table S6. Primers used in this study. [file 12934_2015_288_MOESM1_ESM.docx]

# Supplementary Information

**CrEdit: CRISPR mediated multi-loci gene integration in *Saccharomyces cerevisiae***

Ronda, C.*, Maury J.*, Jakociunas T.*, Baallal Jacobsen S.A., Germann S.M., Harrison S., Borodina I., Keasling J.D., Jensen M.K., Nielsen A.T. ^§^

The Novo Nordisk Foundation Center for Biosustainability, Technical University of Denmark, Kogle Allé 6, 2970 Hørsholm, Denmark

* These authors contributed equally to this work

^§^ Corresponding author

Email addresses:

ATN: [atn@biosustain.dtu.dk](mailto:atn@biosustain.dtu.dk)

# Supplementary Figures

**
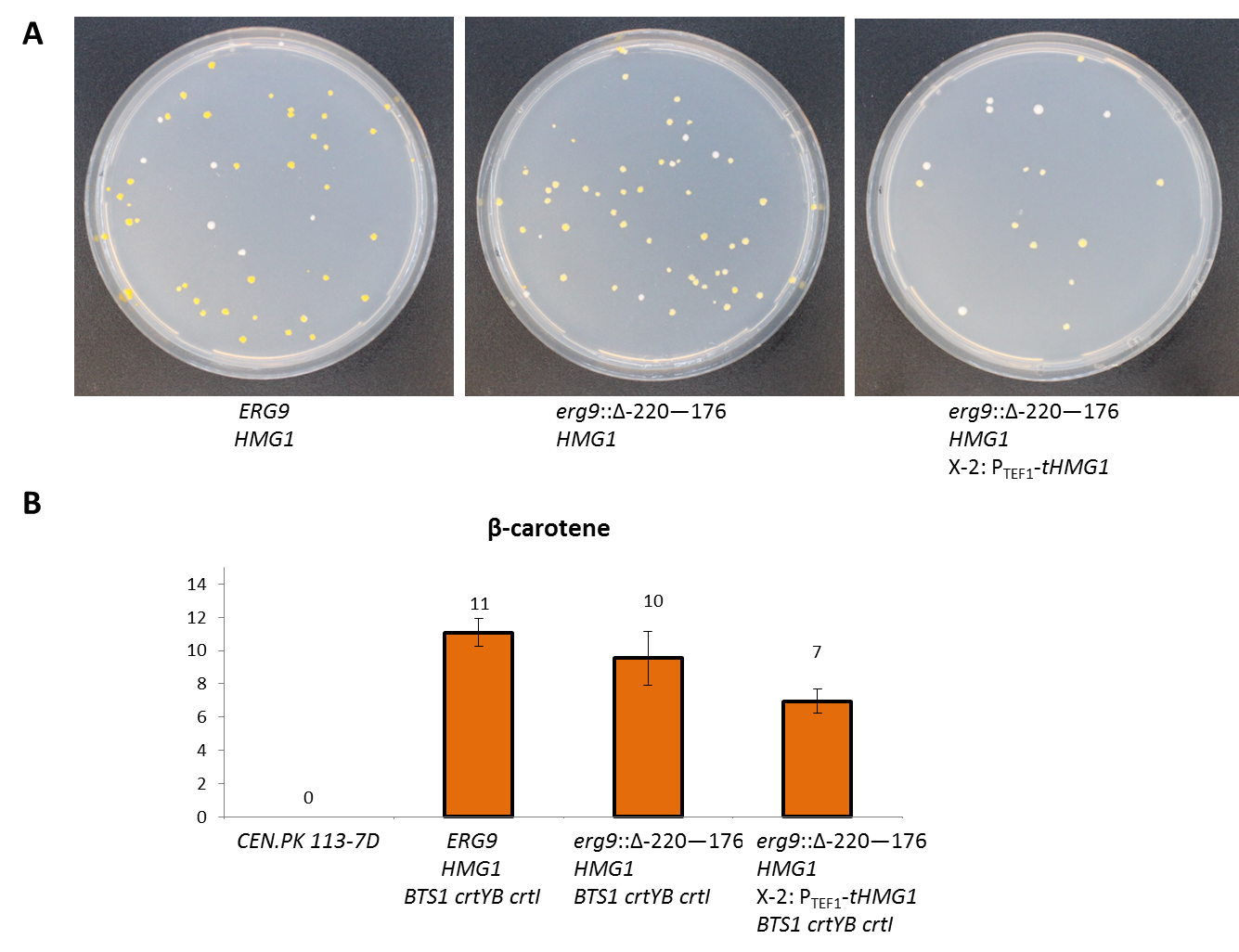
**

**Supplementary Figure S1.** **Efficient single-step integration of the beta-carotenoid pathway.** A) Photos of colonies on plates without selection after simultaneous integration of *BTS1, crtYB* and *crtI* in three different strains. Colonies with successful pathway integration accumulate beta-carotene, resulting in an orange pigmentation. The following three strains, *S. cerevisiae* (TC-3), *erg9*::Δ-220--176 (TC-23), and *erg9*::Δ-220--176 P_TEF1_-*tHMG1* (SCE-iL-CRISPR-8), all harboring a centromeric plasmid constitutively expressing Cas9 (pCfB1767), were co-transformed with triple gRNAs (pTAJAK-92) and three donor DNAs *(*pTAJAK-12, pTAJAK-94, pTAJAK-95). B) Measurement of β–carotene by HPLC analysis of selected orange colonies. Strains analysed are derived from *S. cerevisiae* TC-3 and bear either i) *BTS1, crtYB* and *crtI,* ii) *erg9*::Δ-220—176, *BTS1, crtYB* and *crtI or* iii) *erg9*::Δ-220--176 P_TEF1_-*tHMG1, BTS1, crtYB* and *crtI.* A reference measurement from a strain devoid of the carotenoid pathway, CEN.PK 113-7D, is also shown.

# Supplementary Methods

## Construction of yeast expression plasmids used as donor DNA

The gene fragments (biobricks) carrying the genes and correct overhangs for USER-cloning were generated by PCR amplification using primers and templates as indicated in Supplementary Table S5. The PCR mix contained: The PCR mix contained: 18 µl water, 10 µl HF phusion buffer (5x, BioLab), 5 µl 2mM dNTP, 2 µl PfuX7 polymerase [1, 2], 2.5 µl forward primer ([1]10 µM), 2.5 µl reverse primer (10 µM), and 1 µl DNA template. The PCR program was: 95°C for 2 min, 30 cycles of [95°C for 10 sec, 52°C for 20 sec, 68°C for (1min/kb)], 68°C for 5 min, pause at 10°C. The gene fragments were *Dpn*I-treated, and resolved on 1% agarose gel containing SYBR®-SAFE (Invitrogen) and purified using NucleoSpin® Gel and PCR Clean-up kit (Macherey-Nagel). The promoter fragments were also generated by PCR followed by DNA purification (Supplementary Table S5). The terminators were already present on the yeast vectors. The expression plasmids were created by USER-cloning as described previously using *E. coli* strain DH5alpha [1]. The clones with correct inserts were identified by colony PCR and the plasmids of 4 clones/transformation were isolated from overnight *E. coli* cultures and confirmed by sequencing (Eurofins). The expression plasmids are listed in Supplementary Table S3, and the primers used are listed in Supplementary Table S6. Construction of plasmid pCfB2996 was mainly performed on the Hamilton Vantage Cloning Robot.

For cloning of pTAJAK-94, the backbone vector pCfB257 was amplified using primers TJOS-89F/TJOS-89R and re-ligated by USER ligation in order to mutate the PAM site, resulting in pCfB3422.

# Supplementary Tables

**Supplementary Table S1. Efficiency of targeted integration using CrEdit**

1. **Efficiency of integration at a single site.**

| **Genomic expression of cas9 and gRNA, single integration** | | | | |
| --- | --- | --- | --- | --- |
|  |  | **Selection for donor marker** | **No selection for donor marker** | |
| gRNA | HR arm size (bp) | Integration efficiency at X-2 | URA3-positive clones | Integration efficiency at X-2 |
| - | 500 | 70% (n=64) | 3% (n=640) | 75% (n=16) |
|  | 110 | 0% (n=16) | 0% (n=560) | 0% (no viable colonies) |
|  | 60 | 19% (n=16) | 0% (n=868) | 0% (no viable colonies) |
|  |  |  |  |  |
| + | 500 | 98% (n=64)* | 19% (n=1308)* | 100% (n=16)ns |
|  | 110 | 100% (n=16)* | 3% (n=1552)* | 100% (n=16)NA |
|  | 60 | 100% (n=16)* | 9% (n=1632)* | 100% (n=16)NA |
|  |  |  |  |  |
| **Plasmid-based expression of cas9 and gRNA, single integration** | | | | |
|  |  | **Selection for donor marker** | **No selection for donor marker** | |
| gRNA | HR arm size (bp) | Integration efficiency at X-2 | URA3-positive clones | Integration efficiency at X-2 |
| - | 500 | 100% (n=16) | 0% (n=468) | 0% (no viable colonies) |
|  | 110 | 0% (no viable colonies) | 0% (n=1088) | 0% (no viable colonies) |
|  | 60 | 0% (no viable colonies) | 0% (n=800) | 0% (no viable colonies) |
|  |  |  |  |  |
| + | 500 | 100% (n=15)NA | 99% (n=87)* | 100% (n=16)NA |
|  | 110 | 100% (n=16)NA | 90% (n=20)* | 100% (n=16)NA |
|  | 60 | 100% (n=15)NA | 98% (n=50)* | 100% (n=16)NA |

1. **Efficiency of integration at multiple sites.**

| **Plasmid-based expression of cas9 and gRNA,- multiplex integration** | | | | |
| --- | --- | --- | --- | --- |
|  |  | **No selection for donor marker** | | |
| gRNA | HR arm size (bp) | orange colonies | URA3/HIS5/LEU2-positive  clones | Integration efficiency at  X-3, XI-2 and XII-5 |
| - | 500  500 | 0% (n=351) | 0% (n=351) | 0% (no viable colonies) |
| + |  | 84% (n=101)* | 84% (n=101)* | 100% (n=32)NA |

n = number of colonies analyzed;

Significance between the same sized HR arms, + vs - gRNA: ns = not significant (*P* > 0.05), * = significant (*P* <0.05), NA = not analyzed; The significance of differences in successful integration event numbers was calculated using Student’s t-test.

# Supplementary Table S2: List of strains used in this study.

The strains were constructed by transforming plasmids into yeast chassis (parent strains).

| **Strain name** | **Parent strain (chassis)** | **Added plasmid** | **Relevant genotype*** | **Reference/**  **Source** |
| --- | --- | --- | --- | --- |
| CEN.PK113-7D | - | - | *MAT*a *URA3 HIS3 LEU2 TRP1 MAL2-8^c^ SUC2* | Peter Kötter |
| CEN.PK102-5B | - | - | *MAT*a *ura3-52 his3 Δ 1 leu2-3/112* *MAL2-8c SUC2*  [ura^-^ his^-^ leu^-^] | Peter Kötter |
| CEN.PK2-1C | - | - | *MAT*a *ura3-52 his3 Δ 1 leu2-3/112* *trp1-289 MAL2-8c SUC2* [ura^-^ his^-^ leu^-^ trp^-^] | Peter Kötter |
| ST1011 | CEN.PK102-5B | pCfB1129 | P_CUP1_-Cas9 *loxP-SpHIS5* [ura^-^ leu^-^] | This study |
| TC-3 (ST2148) | CEN.PK2-1C | pCfB1767 | (P_TEF1_-Cas9 *TRP1*)* [ura^-^ his^-^ leu^-^] | [3] |
| TC-23 (ST3007) | CEN.PK2-1C | pCfB1767,  erg9::Δ-220--176 | (P_TEF1_-Cas9 *TRP1*)* erg9::Δ-220--176 [ura^-^ his^-^ leu^-^] | [3] |
| SCE-iL-CRISPR-7  (ST3449) | TC-3 | pCfB2996 | (P_TEF1_-Cas9 *TRP1*)* P_TEF1_-*tHMG1* NatMXsyn  [ura^-^ his^-^ leu^-^ ClonNat^R^] | This study |
| SCE-iL-CRISPR-8  (ST3450) | TC-23 | pCfB2996 | (P_TEF1_-Cas9 *TRP1*)* erg9::Δ-220--176 P_TEF1_-*tHMG1* NatMXsyn [ura^-^ his^-^ leu^-^ ClonNat^R^] | This study |

* (2µ) refers to an episomal high-copy plasmid with 2µ origin.

# Supplementary Table S3: List of plasmids used in this study.

Expression plasmids were constructed by assembling parent plasmid and BioBricks. The resulting relevant gene content, integration site or replicon type and selection maker are shown. All plasmids carry the *AMP^R^* marker for amplification in *E. coli*.

| **Plasmid name** | **Parent plasmid, BioBricks** | **Gene content** | **Integration site/replicon** | **Selection marker** | **Reference/**  **Source** |
| --- | --- | --- | --- | --- | --- |
| pESC-LEU  (pCfB24) | - | - | 2µ, episomal | KlLEU2 | Agilent |
| pSP-GM1  (pCfB29) | - | P_TEF1_, P_PGK1_ | 2µ, episomal | URA3 | [4] |
| pCfB255 | - | USER cloning site | X-2 | loxP-KlURA3 | [1] |
| pCfB257 | - | USER cloning site | X-3 | loxP- KlLEU2 | [1] |
| pCfB258 | - | USER cloning site | X-4 | loxP-SpHIS5 | [1] |
| pCfB261 | \| - \|  \| \| --- \| --- \| | USER cloning site | XII-5 | loxP-SpHIS5 | [1] |
| pCfB389 | - | USER cloning site | XII-2 | loxP-KlURA3 | [1] |
| pCfB772 | pCfB255, tHMG1<-, ScP_TEF1_ | P_TEF1_-*tHMG1* | X-2 | loxP-KlURA3 | This study |
| pJ607-03  (pCfB1183) | - | P_CMV_*-*Cas9 | pUC ori | Hygromycin | DNA2.0 |
| pCfB1129 | pCfB258, SpCas9->, ScP_CUP1_ | P_CUP1_*-* Cas9*-*T_CYC1_ | X-4 | loxP-SpHIS5 | This study |
| YEplac195-crtYB-crtI-BTS1 (pCfB 1764) | - | P_GPD_-ScBTS1  P_GPD_-XdcrtI  P_GPD_-XdcrtYB | 2µ, episomal | KlURA3 | [5] |
| pRS414-Cas9  (pCfB1767) | - | P_TEF1_-Cas9-T_CYC1_ | *ARS/CEN* | ScTRP1 | [6]  (Addgene reference number: 43802) |
| pCfB1954  (pTAJAK-12) | pCfB261, BTS1->, ScP_PGK1_ | P_PGK1_-*BTS1* | XII-5 | loxP-SpHIS5 | This study |
| pCfB2179 | **-** | P_AgTEF1_-KanMXsyn | - | loxP-KanMXsyn | GeneArt |
| pCfB2180 | - | P_AgTEF1_-NatMXsyn | - | loxP-NatMXsyn | GeneArt |
| pCfB2831 | pCfB257, gBlock_X-2’ | P_SNR52_-gRNA_X-2 | X-3 | loxP-KlLEU2 | This study |
| pCfB2193 | - | USER cloning site | X-2 | loxP-NatMXsyn | [7] |
| pCfB2926 (pTAJAK-71) | pESC-LEU, see materials and methods for detailed cloning | USER cloning site | 2µ, episomal | loxP-NatMXsyn | This study |
| pCfB2996 | pCfB2193, tHMG1<-, ScP_TEF1_ | P_TEF1_-*tHMG1* | X-2 | loxP-NatMXsyn | This study |
| pCfB2999  (pTAJAK-72) | pESC-LEU, see materials and methods for detailed cloning | USER cloning site | 2µ, episomal | loxP-KanMXsyn | This study |
| pCfB3020 (pTAJAK-76) | pCfB2926, gBlock_X-2 | P_SNR52_-gRNA_X-2 | 2µ, episomal | loxP-NatMXsyn | This study |
| pCfB3065  (pTAJAK-92) | pCfB2926, gBlock_X-3, gBlock_XI-2, gBlock_XII-5 | P_SNR52_-gRNA_X-3_XI-2_XII-5 | 2µ, episomal | loxP-NatMXsyn | This study |
| pCfB3067  (pTAJAK-94) | pCfB257mut, crtI<-, ScP_TDH3_ | P_TDH3_-crtI | X-3 | loxP-KlLEU2 | This study |
| pCfB3068  (pTAJAK-95) | pCfB389, crtYB->, ScP_TEF1_ | P_TEF1_-crtYB | XI-2 | *loxP-KlLEU2* | This study |
| pCfB3422 | pCfB257, PAM mutated | USER cloning site | X-3 | loxP- KlLEU2 | This study |

# Sequence Listing

IDT-gBlocks were build according to the basic structural principle published previously [6]. Shown in Supplementary Table S4 are common features of all gBlocks, the *SNR52* promoter, the synthetic scaffold and the *SUP4* terminator. The underlined region in the SEQ IDs corresponds to the target sequence.

# Supplemental Table S4: gRNA components common to all used gBlocks


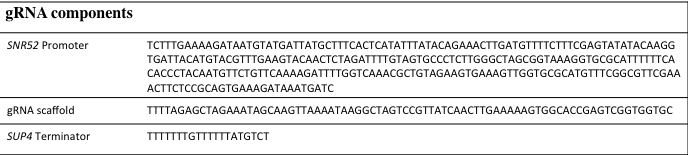


SEQ ID NO: 1

gRNA_X-2’

TCTTTGAAAAGATAATGTATGATTATGCTTTCACTCATATTTATACAGAAACTTGATGTTTTCTTTCGAGTATATACAAGGTGATTACATGTACGTTTGAAGTACAACTCTAGATTTTGTAGTGCCCTCTTGGGCTAGCGGTAAAGGTGCGCATTTTTTCACACCCTACAATGTTCTGTTCAAAAGATTTTGGTCAAACGCTGTAGAAGTGAAAGTTGGTGCGCATGTTTCGGCGTTCGAAACTTCTCCGCAGTGAAAGATAAATGATCCTCTCGAAGTGGTCACGTGCTTTTAGAGCTAGAAATAGCAAGTTAAAATAAGGCTAGTCCGTTATCAACTTGAAAAAGTGGCACCGAGTCGGTGGTGCTTTTTTTGTTTTTTATGTCT

SEQ ID NO: 2

gRNA_X-2

agggaacaaaagctggagctTCTTTGAAAAGATAATGTATGATTATGCTTTCACTCATATTTATACAGAAACTTGATGTTTTCTTTCGAGTATATACAAGGTGATTACATGTACGTTTGAAGTACAACTCTAGATTTTGTAGTGCCCTCTTGGGCTAGCGGTAAAGGTGCGCATTTTTTCACACCCTACAATGTTCTGTTCAAAAGATTTTGGTCAAACGCTGTAGAAGTGAAAGTTGGTGCGCATGTTTCGGCGTTCGAAACTTCTCCGCAGTGAAAGATAAATGATCctctcgaagtggtcacgtgcGTTTTAGAGCTAGAAATAGCAAGTTAAAATAAGGCTAGTCCGTTATCAACTTGAAAAAGTGGCACCGAGTCGGTGGTGCTTTTTTTGTTTTTTATGTCTtcgagtcatgtaattagtta

SEQ ID NO: 3

gRNA_X-3

agggaacaaaagctggagctTCTTTGAAAAGATAATGTATGATTATGCTTTCACTCATATTTATACAGAAACTTGATGTTTTCTTTCGAGTATATACAAGGTGATTACATGTACGTTTGAAGTACAACTCTAGATTTTGTAGTGCCCTCTTGGGCTAGCGGTAAAGGTGCGCATTTTTTCACACCCTACAATGTTCTGTTCAAAAGATTTTGGTCAAACGCTGTAGAAGTGAAAGTTGGTGCGCATGTTTCGGCGTTCGAAACTTCTCCGCAGTGAAAGATAAATGATCctAAtgtgtccgcgtttctaGTTTTAGAGCTAGAAATAGCAAGTTAAAATAAGGCTAGTCCGTTATCAACTTGAAAAAGTGGCACCGAGTCGGTGGTGCTTTTTTTGTTTTTTATGTCTtcgagtcatgtaattagtta

SEQ ID NO: 4

gRNA_XI-2

agggaacaaaagctggagctTCTTTGAAAAGATAATGTATGATTATGCTTTCACTCATATTTATACAGAAACTTGATGTTTTCTTTCGAGTATATACAAGGTGATTACATGTACGTTTGAAGTACAACTCTAGATTTTGTAGTGCCCTCTTGGGCTAGCGGTAAAGGTGCGCATTTTTTCACACCCTACAATGTTCTGTTCAAAAGATTTTGGTCAAACGCTGTAGAAGTGAAAGTTGGTGCGCATGTTTCGGCGTTCGAAACTTCTCCGCAGTGAAAGATAAATGATCGTTGACCAGTTGATCAGTTGGTTTTAGAGCTAGAAATAGCAAGTTAAAATAAGGCTAGTCCGTTATCAACTTGAAAAAGTGGCACCGAGTCGGTGGTGCTTTTTTTGTTTTTTATGTCTtcgagtcatgtaattagtta

SEQ ID NO: 5

gRNA_XII-5

agggaacaaaagctggagctTCTTTGAAAAGATAATGTATGATTATGCTTTCACTCATATTTATACAGAAACTTGATGTTTTCTTTCGAGTATATACAAGGTGATTACATGTACGTTTGAAGTACAACTCTAGATTTTGTAGTGCCCTCTTGGGCTAGCGGTAAAGGTGCGCATTTTTTCACACCCTACAATGTTCTGTTCAAAAGATTTTGGTCAAACGCTGTAGAAGTGAAAGTTGGTGCGCATGTTTCGGCGTTCGAAACTTCTCCGCAGTGAAAGATAAATGATCttgtcacagtgtcacatcagGTTTTAGAGCTAGAAATAGCAAGTTAAAATAAGGCTAGTCCGTTATCAACTTGAAAAAGTGGCACCGAGTCGGTGGTGCTTTTTTTGTTTTTTATGTCTtcgagtcatgtaattagtta

# Supplementary Table S5: DNA BioBricks and gBlocks.

Genes, promoters (BioBricks) and gBlocks were PCR-amplified from the indicated templates using the corresponding forward and reverse oligos.

| **BioBrick name*** | **Description** | **Oligo forward** | **Oligo Reverse** | **Template** |
| --- | --- | --- | --- | --- |
| <-ScP_TEF1_ | Promoter of *TEF1* gene (*S. cerevisiae*) | PR-5 | PR-6 | pSP-GM1 |
| ->ScP_PGK1_ | Promoter of *PGK1* gene (*S. cerevisiae*) | PR-7 | PR-8 | pSP-GM1 |
| ->ScP_TEF1_ | Promoter of *TEF1* gene (*S. cerevisiae*) | PR-1564 | PR-1565 | pSP-GM1 |
| <-ScP_TDH3_ | Promoter of *TDH3* gene (*S. cerevisiae*) | PR-1852 | PR-1853 | Genomic DNA of WT CEN.PK |
| ->ScP_CUP1_ | Promoter of *CUP1* gene (*S. cerevisiae*) | PR-1757 | PR-1758 | Genomic DNA of WT CEN.PK |
| SpCas9-> | CRISPR associated protein 9 gene (*S. pyogenes*) | PR-10733 | PR-10734 | pJ607-03 (DNA2.0) |
| tHMG1<- | Truncated HMG-CoA reductase gene (*S. cerevisiae*) | PR-1321 | PR-1322 | Genomic DNA of WT CEN.PK |
| BTS1-> | geranylgeranyl diphosphate (GGPP) synthase gene (*S. cerevisiae*) | PR-7045 | PR-7046 | YEplac195-crtYB-crtI-BTS1 |
| crtI<- | phytoene desaturase gene (*X. dendrorhous*) | PR-7041 | PR-7042 | YEplac195-crtYB-crtI-BTS1 |
| crtYB-> | bifunctional  phytoene synthase and lycopene cyclase gene (*X. dendrorhous*) | PR-7039 | PR-7040 | YEplac195-crtYB-crtI-BTS1 |
|  |  |  |  |  |
| **gBlock name** | **Description** | **Oligo forward** | **Oligo Reverse** | **Template** |
| gRNA_X-2’ | P_SNR52_-gRNA_X-2’-gRNAscaffold-T_SUP4_ | PR-10737 | PR-11125 | Integrated DNA Technologies (IDT)-gBlock, SEQ ID NO: 1 |
| gRNA_X-2 | P_SNR52_-gRNA_X-2-gRNAscaffold-T_SUP4_ | PR-10525 | PR-10529 | IDT-gBlock, SEQ ID NO: 2 |
| gRNA_X-3 | P_SNR52_-gRNA_X-3-gRNAscaffold-T_SUP4_ | PR-10525 | PR-10530 | IDT-gBlock SEQ ID NO: 3 |
| gRNA_XI-2 | P_SNR52_-gRNA_XI-2-gRNAscaffold-T_SUP4_ | PR-10526 | PR-10531 | IDT-gBlock, SEQ ID NO: 4 |
| gRNA_XII-5 | P_SNR52_-gRNA_XII-5-gRNAscaffold-T_SUP4_ | PR-10527 | PR-10529 | IDT-gBlock, SEQ ID NO: 5 |

* “<-“ indicates gene position 1 and “->” indicates gene position 2 as described in [1].

# Supplementary Table S6: Primer sequences.

Sequences of primers (5´ to 3´) used in this study. Overhangs used for USER cloning are underlined.

| **Primer ID** | **Primer name** | **Primer sequence, 5´ to 3´** |
| --- | --- | --- |
| PR-5 | PTEF1-fw | ACCTGCAC**U** TTGTAATTAAAACTTAG |
| PR-6 | PTEF1-rv | CACGCGA**U** GCACACACCATAGCTTC |
| PR-7 | PPGK1-fw | CGTGCGA**U** GGAAGTACCTTCAAAGA |
| PR-8 | PPGK1-rv | ATGACAGA**U** TTGTTTTATATTTGTTG |
| PR-899 | XII-5-up-out-sq | CCACCGAAGTTGATTTGCTT |
| PR-901 | X-2-up-out-sq | TGCGACAGAAGAAAGGGAAG |
| PR-909 | XI-2-sq-fw | GTTTGTAGTTGGCGGTGGAG |
| PR-1321 | JM166_tHMG1_gene1_fw | AGTGCAGG**U** AAAACAATGACTGCAGACCAATTGGTG |
| PR-1322 | JM136_tHMG1_gene1_rv | CGTGCGA**U** TCAGGATTTAATGCAGGTGACG |
| PR-1564 | PTEF1->_U2_fw | CGTGCGA**U** GCACACACCATAGCTTC |
| PR-1565 | PTEF1->_U2_rv | ATGACAGA**U** TTGTAATTAAAACTTAG |
| PR-1757 | JM197_pCUP1_GENE2_fw | CGTGCGA**U** TTACCGACATTTGGGCG |
| PR-1758 | JM198_pCUP1_GENE2_rev | ATGACAGA**U** TTTATGTGATGATTGATTGATTGATTG |
| PR-1852 | PTDH3_fw | CACGCGA**U** ATAAAAAACACGCTTTTTCAG |
| PR-1853 | PTDH3_rv | ACCTGCAC**U** TTTGTTTGTTTATGTGTGTTTATTC |
| PR-2221 | JM234_ColoPCR_vec_TADH1_towards out | GTTGACACTTCTAAATAAGCGAATTTC |
| PR-7039 | TJOS-23F | ATCTGTCA**U** ATGACGGCTCTCGCATATTA |
| PR-7040 | TJOS-23R | CACGCGA**U** TTACTGCCCTTCCCATCCGC |
| PR-7041 | TJOS-24F | AGTGCAGG**U** ATGGGAAAAGAACAAGATCAGG |
| PR-7042 | TJOS-24R | CGTGCGA**U** TCAGAAAGCAAGAACACCAACG |
| PR-7045 | TJOS-26F | ATCTGTCA**U** ATGGAGGCCAAGATAGATGAG |
| PR-7046 | TJOS-26R | CACGCGA**U** TCACAATTCGGATAAGTGGTCT |
| PR-9704 | 60bp-X-2 UP_Forw (JM275) | TCCATTTCTTTTTCCTCGGGCAG |
| PR-9705 | 60bp-X-2 DW_rev (JM276) | AGTATGATAAATCTTCAGCATAGATGG |
| PR-9706 | 110bp-X-2 UP_Forw (JM277) | CGCATCTATTTGCCGTCAAAAGA |
| PR-9707 | 110bp-X-2 DW_rev (JM278) | GGTTTTCTTTCACGACACACCTCAC |
| PR-10525 | TJOS-62 (P1F) | CGTGCGA**U** AGGGAACAAAAGCTGGAGCT |
| PR-10526 | TJOS-63 (P2F) | AGTGCAGG**U** AGGGAACAAAAGCTGGAGCT |
| PR-10527 | TJOS-64 (P3F) | ATCTGTCA**U** AGGGAACAAAAGCTGGAGCT |
| PR-10529 | TJOS-65 (P1R) | CACGCGA**U** TAACTAATTACATGACTCGA |
| PR-10530 | TJOS-66 | ACCTGCAC**U** TAACTAATTACATGACTCGA |
| PR-10531 | TJOS-67 (P3R) | ATGACAGA**U** TAACTAATTACATGACTCGA |
| PR-11130 | TJOS-89F | ccacttttcaatgaaacgga |
| PR-11131 | TJOS-89R | cttagaaacgcggacacaat |
| PR-11132 | TJOS-97F | GAATGCGTGCGATCGCGTGCATTCcagctgcattaatgaatcgg |
| PR-11133 | TJOS-97R | cagctggcgtaatagcgaag |
| PR-11134 | TJOS-102R | Cccgctgacgcgccctgacg |
| PR-11135 | TJOS-106F | CTGCATTTAAATGATCTGTTTAGCTTGCCTCG |
| PR-11136 | TJOS-106R | CTGCGCGGCCGCAGCTCGTTTTCGACACTGGA |
| PR-11137 | TJOS-108 | CTGCGCGGCCGCctacagggcgcgtcgcgcca |
| PR-10733 | Cas9_fwd | ATCTGTCA**U** AAAACAATGGACAAGAAATACTCCATCGGCCT |
| PR-10734 | Cas9_rev | CACGCGA**U** TCAGACCTTGCGCTTCTTCTTCGG |
| PR-10735 | gRNA_yeast univ _fwd | CGTGCGA**U** TCTTTGAAAAGATAATGTAT |
| PR-10736 | gRNA yeast univ _rev | CACGCGA**U** AGACATAAAAAACAAAAAAA |

# Supplementary References

1. Jensen NB, Strucko T, Kildegaard KR, David F, Maury J, Mortensen UH, Forster J, Nielsen J, Borodina I: **EasyClone: method for iterative chromosomal integration of multiple genes in Saccharomyces cerevisiae**. *FEMS Yeast Res* 2014, **14**:238–248.

2. Nørholm MHH: **A mutant Pfu DNA polymerase designed for advanced uracil-excision DNA engineering.** *BMC Biotechnol* 2010, **10**:21.

3. Jakočiūnas T, Bonde I, Herrgård M, Harrison SJ, Kristensen M, Pedersen LE, Jensen MK, Keasling JD: **Multiplex metabolic pathway engineering using CRISPR/Cas9 in Saccharomyces cerevisiae**. *Metab Eng* 2015.

4. Partow S, Siewers V, Bjørn S, Nielsen J, Maury J: **Characterization of different promoters for designing a new expression vector in Saccharomyces cerevisiae.** *Yeast* 2010, **27**:955–964.

5. Verwaal R, Wang J, Meijnen J-P, Visser H, Sandmann G, van den Berg JA, van Ooyen AJJ: **High-level production of beta-carotene in Saccharomyces cerevisiae by successive transformation with carotenogenic genes from Xanthophyllomyces dendrorhous.** *Applied and Environmental Microbiology* 2007, **73**:4342–4350.

6. DiCarlo JE, Norville JE, Mali P, Rios X, Aach J, Church GM: **Genome engineering in Saccharomyces cerevisiae using CRISPR-Cas systems.** *Nucleic Acids Research* 2013, **41**:4336–4343.

7. Stovicek V, Borodina I, Forster J. :**CRISPR-Cas system enables fast and simple genome editing of industrial *Saccharomyces cerevisiae* strains**. *Submitted*
